# Scaling analysis for electrospinning

**DOI:** 10.1186/2193-1801-3-705

**Published:** 2014-12-02

**Authors:** Siddharth B Gadkari

**Affiliations:** IITB-Monash Research Academy, Indian Institute of Technology Bombay, Powai, 400076 Mumbai, India

**Keywords:** Electrospinning, Scaling analysis, Correlation

## Abstract

Electrospinning refers to the process of generating nanofibers from electrified viscous polymeric jets. Though relatively easy to perform, this process is quite complex in its nature, given the large number of parameters that are involved. This study attempts to derive a relation between the final fiber diameter and the major process parameters. Two new dimensionless numbers describing viscous and surface charge repulsion effects are identified from the scaling analysis of governing equation for the motion of a bent jet. Experimental data for a wide range of polymer solutions exhibit a common slope, when expressed in terms of these new dimensionless numbers. This correlation is used to derive a new scaling expression for the final fiber diameter.

## Introduction

Electrospinning is a simple and versatile method for producing polymeric nanofibers. When high voltage, of the order of kilovolts, is applied between a capillary (or a syringe needle tip) containing polymer solution and a grounded collector, the hemispherical drop at the tip of the needle undergoes transformation into a conical shape, known as the ‘Taylor cone’. On increasing the voltage further, an electrified thin fluid jet is ejected from the tip of the Taylor cone. The jet is initiated once the electrical forces overcome the surface tension, viscoelastic forces, and the interfacial drawing forces from the inner wall of the capillary (Reneker et al. 2000; Yu et al. 2013). This jet follows a straight path for a certain distance but soon succumbs to numerous electrohydrodynamic instabilities. The most dominant among these is the whipping instability which results in rapid chaotic movement of the jet in concentric circles of increasing diameter. This motion results in extensive elongation and causes extreme thinning of the jet. As the jet moves down, it dries & solidifies and gets deposited on the collector (Hohman et al. 2001).

While electrospinning is quite a simple process to perform, the properties of the final fiber depend on a multitude of molecular, process, and operational parameters. It involves a complex interplay between fluid dynamics, electrodynamics and rheology. Given this complexity, it is difficult to derive a mathematical model for the complete process. In fact most of the current models available are either limited to the steady jet region or are too complex for numerical solutions (Feng 2002; Hohman et al. 2001; Reneker et al. 2000). Although the double/triple-fluid electrospinning processes are broadly reported in literature (Han and Steckl 2009; McCann et al. 2006; Yarin 2011; Yu et al. 2011; Zhang et al. 2004), the complete mechanism about a single-fluid electrospinning process is still not clear.

Simple expressions that could relate the process parameters to the final fiber properties could be great help in analysing the electrospinning process. Such a predictive tool will help to verify and understand many different polymer-solvent systems and guide the experimental studies. There are some correlations reported in literature to foresee a priori the properties of the final electrospun fibers, given the operating parameters and starting solution properties (Fridrikh et al. 2003; Helgeson and Wagner 2007; Helgeson et al. 2008). However each one of these correlations have their own limitations and are mostly limited to selective polymer-solvent systems.

In this study, an attempt to find a relation between final fiber diameter and the important process parameters has been made. The paper is organized as follows, first a literature review on various correlations is presented, followed by the asymptotic analysis and finally the correlation between the two new dimensionless numbers is presented.

### Experimental observations

Many researchers have performed detailed investigations to study the effect of the several key parameters on the diameter of electrospun fibers (Beachley and Wen 2009; Cui et al. 2007; Deitzel et al. 2001; Greiner and Wendorff 2008). From experimental investigations reported in literature, solution viscosity, flow rate and applied voltage can be considered as the three most important parameters affecting the final fiber diameter and morphology. Some other parameters such as tip-to-collector distance, solution conductivity and surface tension, have relatively less influence (Ramakrishna et al. 2005).

There is a general agreement in literature that the solution viscosity and applied flow rate are directly proportional to the final fiber diameter *d*_*f*_, i.e. *d*_*f*_ increases with increase in zero shear rate viscosity *η*_0_ and applied flow rate *Q*. In some studies the authors have also reported the scaling coefficient. For example, the scaling coefficient for viscosity has been shown to shown to vary in the range of 0.4–0.7 (Gupta et al. 2005; McKee et al. 2004; Wang et al. 2008).

While there are many studies which reported that *d*_*f*_ increases with increasing flow rate, only few of them have shown systematic data which gives scaling coefficient between the two. Experimental data provided by Wang et al. (2008), Liu et al. (2007) and Mataram et al. (2011) was used to extract the scaling coefficient for *Q* as 0.3, 0.34 and 0.35 respectively.

The relation between applied voltage △*V* and fiber diameter is ambiguous. Even though majority of the studies have found that the fiber diameter decreases with increasing voltage (Homayoni et al. 2009; Liu et al. 2007; Mataram et al. 2011; Mazoochi and Jabbari 2011; Sajeev et al. 2008; Thompson et al. 2007; Wang et al. 2009; Yuan et al. 2004; Zhang et al. 2005), there are some experimental studies which have observed an inverse relation (Jeun et al. 2007; Rojas et al. 2009). There are some other studies which have reported a dual effect of applied voltage, in which either the fiber diameter first decreases with increasing voltage up to a threshold value and then increases with further increase in voltage (Supaphol and Chuangchote 2008; Wannatong et al. 2004) or vice-versa (Singh et al. 2009). This ambiguous relation between *d*_*f*_ and △*V* is probably because △*V* is interconnected with tip-to-collector distance *L*, as their ratio (△*V*/*L*) determines the applied electric field on the jet. The increase in applied electric field (△*V*/*L*) can increase the drawing forces for finer nanofibers, but meanwhile it also promote the faster evaporation of solvents and solidification of nanofibers to let the electrical forces lose efficacy. Similarly many other parameters such as solution concentration and conductivity also influence the overall effect of the applied voltage or the electric field on fiber diameter (Mohan 2002; Thompson et al. 2007). These interlinks with other parameters make it difficult to analyze the individual effect of applied voltage.

There are very few studies where effect of conductivity *K* and surface tension *γ* has been investigated independently. Zhang et al. (2005) observed a decrease in fiber diameter with increase in conductivity of the polymer solution whereas Kim et al. (2005) and Mituppatham et al. (2004) reported a reverse trend.

The experimental observations discussed above present the immense complexity of the electrospinning process. The final fiber properties not only depend on a large number of parameters, the scaling with each parameter also depends on the type of polymer-solvent system used. All these factors present a significant challenge to derive one universal correlation for the fiber diameter. Some of the correlations proposed so far, are discussed in the next section.

### Existing empirical and semi-empirical correlations

Fridrikh et al. (2003) presented a model of a charged Newtonian fluid jet in an electric field under conditions applicable to whipping instability. The model predicts that the final diameter of the jet arises from a balance between surface charge repulsion and surface tension forces. Here they have used the equation of motion for normal displacements *X* of the centerline of a bent jet, based on force and angular momentum balance, derived previously by Hohman et al. (2001).

Fridrikh et al. (2003) derived the following relation for the terminal jet diameter, *h*_*t*_:
1

where, *γ* is the surface tension,  is the dielectric permittivity of atmosphere and *I* is the total current.

Using the above expression for terminal diameter, Fridrikh et al. (2003) compared experimental data for dry fiber diameters obtained using polycaprolactone (PCL), polyethylene oxide (PEO) and polyacrylonitrile (PAN) polymer solutions with the theoretically predicted values. Quantitative agreement between observed and predicted fiber diameters, however, was found in some, but not all cases. PAN and PEO data showed good agreement, but the model over-predicted stretching for PCL. Fridrikh et al. (2003) attributed the difference in charge carriers and solvent to explain the discrepancy in the agreements. It should be noted that Eq. (1) does not account for solution viscosity, polymer contribution and solvent evaporation. This leads to an inconsistently large role being attributed to surface tension, which based on experimental observations is one of the least important factor in determining fiber diameter.

Helgeson and Wagner (2007) derived an empirical correlation for fiber diameter using two new dimensionless numbers obtained by combining existing non-dimensional numbers that were previously defined by Feng (2002) in his analysis of steady state electrospinning jet. The two new dimensionless numbers were, , representing the strength of electrostatic stress relative to electro-viscous stress and *Π*_2_=(*ργR*_*jet*_)/*η*_0_=*Oh*^−2^, representing the ratio of inertial to viscous forces. Here *ρ* is the density of the polymer solution, *E*_0_ is the applied electric field and *R*_*jet*_ represents wet radius of the electrospinning jet, and *R*_*jet*_=*h*_*t*_/2.

Helgeson and Wagner (2007) found that values of *Π*_1_ and *Oh* from several data sets for different polymer-solvent systems when plotted on one plot reduced onto a single master curve. Two empirical trends were observed in the master plot, first at large *Oh* where an inverse relationship was observed, such that , and the other regime at smaller values of *Oh*, where the scaling shifted to −3/4 slope from −1.

The scaling at large *Oh* resulted in the following expression,
2

Helgeson and Wagner (2007) proposed that by using Eq. (2), it is possible to get an estimate of the final fiber diameter only based on surface tension, solution conductivity, density and applied electric field values. As *E*_0_ was defined as, *E*_0_=△*V*/*L*, this model predicts an increase in fiber radius with increasing applied voltage (△*V*). While there are some experimental studies which predict a increase in final fiber diameter with △*V* (Jeun et al. 2007; Rojas et al. 2009), there are several other studies which predict an inverse scaling between the two (Liu et al. 2007; Sajeev et al. 2008; Thompson et al. 2007; Wang et al. 2009; Zhang et al. 2005).

In a follow-up paper, Helgeson et al. (2008) identified a new dimensionless number,
3

using asymptotic analysis in the jet stretching regime.

Here, *ε* represents the permittivity of the electrospinning fluid,  is the steady state extensional viscosity, *r*_*f*_ represents the radius of the final electrospun fiber. *r*_*f*_ and *R*_*jet*_ are related as, *R*_*jet*_=*r*_*f*_∗(*c*)^−0.5^. *c* is the polymer solution concentration.

When Helgeson et al. (2008) plotted data for PEO-water and PEO-NaCL-Water system from experiments for *Oh*_*f*_ (as defined previously by Helgeson and Wagner (2007)) and *Π*_*f*_, they obtained the following scaling between the two, . This resulted in the following empirical relationship for the final fiber diameter:
4

While calculating the value of *Π*_*f*_, in place of , Helgeson et al. (2008) have used 3*η*_0_. This results in the following scaling for viscosity, . This scaling does not agree with any of the given correlations in literature. Also, Eq. (4) has been shown to be valid only for PEO-water solution, and for no other polymer-solvent system. For PEO-NaCl-water system, there exists a different scaling, namely , which was attributed to the influence of bending instability. This suggests that scaling predicted by Helgeson et al. (2008) between fiber diameter and process parameters is not universal and thus may not be very useful for different polymer-solvent systems.

After reviewing all the above correlations, it can be concluded that, even though progress has been occasionally achieved in some specific cases, research is still far from one universal correlation for predicting the final electrospun fiber diameter. This is probably due to the poor understanding of the complex whipping instability region and the number of inter-related variable interactions occurring during the electrospinning process.

## Scaling analysis

Hohman et al. (2001) proposed the equation of motion for the normal displacements of the centerline of the bent jet, which is observed at the onset of whipping. The full equation is quite complicated and involves many variables that are difficult to measure. Usually when flow problems are too complex for analytical or numerical solution, it is a standard engineering practice to non-dimensionalize the governing equations and the non-dimensional numbers thus obtained are then used to guide experimental data correlations.

Hohman et al. (2001) non-dimensionalized the electrohydrodynamic governing equations valid for the jet region of electrospinning before whipping starts, using the following scaling, radius of the capillary or the needle tip *r*_*c*_ for both jet radius *h* and *z*,  for time,  for electric field and  for surface charge density.

The diameter of the jet at the onset of whipping or in the terminal steady jet regime is about 3−4 orders of magnitude less than the nozzle radius, thus it is not appropriate to continue using the same scaling for the jet radius. In this study, a revised scaling for the non-dimensionalization of the equation for bent jet is proposed and the new dimensionless numbers obtained are examined.

### Asymptotic analysis

Feng (2002) proposed the governing equations for the steady jet region of electrospinning by considering the conservation of mass, conservation of charge, conservation of momentum and the electric field variation along the jet. These governing equations, which were only limited to Newtonian solutions are as follows,
5678

where, *v* is the fluid velocity parallel to the jet and prime indicates derivatives with respect to *z*. Feng (2002) used the following scaling for non-dimensionalisation of the above governing equations: *r*_*c*_ for both *h* and *z*,  for velocity,  for electric field and  for surface charge density.

In the terminal steady jet region, the tangential electrical forces (2*σE*/*h*) dominate the acceleration of the jet and thus near the end of the steady jet region, the governing balance in Eq. (7) is reduced to,
9

Also, the convective current dominates the major contribution to the total current in the steady jet region, thus
10

Using Equations (5), (9) and (10), we get,
1112

The above expression correctly predicts the radius of the jet in the terminal region of the steady jet. Similar scaling expression (*h*∝*z*^−1/4^) has been previously derived by Kirichenko et al. (1986) for an inviscid jet.

### Revised scaling

Radius of the jet further downstream of the terminal steady jet is comparable to that obtained from Eq. (12). Thus this radius could be used as the new characteristic scale for radius further downstream in the whipping zone. *z* in Eq. (12) can be replaced by *L*. This new characteristics scaling for radius of the jet (*h*) as *R*_*om*_.
13

However in the whipping region, scaling for *h* and *z* cannot be same anymore because *z* is about 3−4 orders of magnitude more than *R*_*om*_. *L*, the total distance between needle tip and collector as the new characteristic scale for *z*.

The corresponding new scale for velocity is,
14

Also, the previous scaling for electric field  is no longer suitable and the applied electric field, *ΔV*/*L*, is used as the new characteristic scaling for *E*.

As the surface charge convection is the main contributor to current in the slender jet, the new characteristic scale for *σ* is chosen as,
15

Current *I* is usually not measured in most of the experimental electrospinning studies. In such situations many a time the non-measurable quantity is expressed in terms of other measurable quantities. In this analysis, the recently proposed scaling for current in electrospinning by Bhattacharjee et al. (2010) is used:
16

After substituting for electric field and current, the revised scale for radius (*R*_*om*_) is,
17

For radius of curvature of whipping (*R*_*c*_), *L* is used and the normal displacement of the centerline (*X*) and arc length of the jet (*s*) are scaled using *r*_*c*_. For time *t*, the viscous time scale *τ*_*vis*_=6*η*_0_*L*/*γ*, is used for non-dimensionalisation.

### Non-dimensionalisation

The full equation for the force balance of a bent jet, considering both electric field and surface charge, as derived by Hohman et al. (2001) is given as,
18

where the coefficients are defined as,


Here *ρ* is the density of the polymer solutions, *h* radius of the jet, *R*_*c*_ radius of curvature of whipping, *s* arc length of the jet, *σ* surface charge density, *E* electric field, *ξ* static charge density, *γ* surface tension, *ε* fluid dielectric constant,  air dielectric constant, , *K* conductivity, *k* wavenumber of the instability.

After substituting the above scaling in the full force balance on a bent jet (Eq. 18), the following non-dimensional form with 10 new dimensionless numbers is obtained, represented by *Π*_*i*_ with *i*=1,2…,10.
19

where,


### Correlation between dimensionless numbers

Among the ten dimensionless numbers, the only term which inherently involved viscosity was *Π*_7_, derived from the viscous moment contribution in *A*_3_. As discussed before, experimental observations suggest a strong correlation between final fiber diameter and the viscosity of the pre-cursor polymer solution. The viscous moment in *A*_3_ helps to stabilize the jet against the destabilizing action of surface charge repulsion (*B*_4_). The dimensionless number corresponding to *B*_4_ was *Π*_9_. To understand if these two dimensionless numbers had an influence on the final fiber diameter, *Π*_7_ was plotted against *Π*_9_ using experimental data from literature for several different polymer-solvent systems.

Experimental data compiled in Table 1 represents a wide range of solution properties and operating parameters. To include the effect of fiber diameter, *σ*_*om*_ term in *Π*_9_ was calculated using *d*_*f*_ instead of *R*_*om*_ such that, *σ*_*om*_=*d*_*f*_*I*/2 *Q*. This modified *Π*_9_ was called as .

**Table 1:** **Experimental data collected from literature to be used in the analysis**

Polymer-solvent system	Cited work	***η***	K	***γ***	***Δ*** ***V***	***L***	***Q***	***d***_***f***_
		( ***P*** ***a*** ***s***)	( ***S***/ ***m***)	( ***N***/ ***m***)	( ***K*** ***V***)	( ***c*** ***m***)	***m*** ***L***/ ***h*** ***r***	( ***n*** ***m***)
PS/THF/LiCI *O*_4_	Wang et al. (2006)	0.2–2.5	0.00012	0.0242	10	14	3	2400–7400
DNA/PEO/Water	Liu et al. (2007)	2.5	0.064	0.042	5–20	10–25	6	130–210
PA-6/FA	Mit-uppatham et al. (2004)	1.2–4.6	0.04–0.4	0.04–0.044	21	10	3	90–200
PA-6/FA/DCM	Wei et al. (2010)	0.2–14	0.13–0.45	0.035–0.045	20	20	0.2	80–550
PS-U2	Wang et al. (2011)	0.025–0.1	0.0002–0.0003	0.0358	5	14	1	200–1000
PAN/DMF	Wang et al. (2007)	0.2–1.5	0.0035–0.0046	0.0363	6	7	0.3	250–550
PHB/CF/DMF	Wang et al. (2008)	0.1–5	0.00025	0.028	10	14	5–14	950–5700
PAA-Water	Li and Hsiehg (2005)	0.03–0.7	0.002–0.02	0.043	13.5	25	1	90–550
PAA-DMF	Li and Hsiehg (2005)	0.1–4.0	0.00006–0.00007	0.038	5	25	1	60–330

PS-U2: PS of MW 1.88***∗***10^6^.

**Figure 1:**
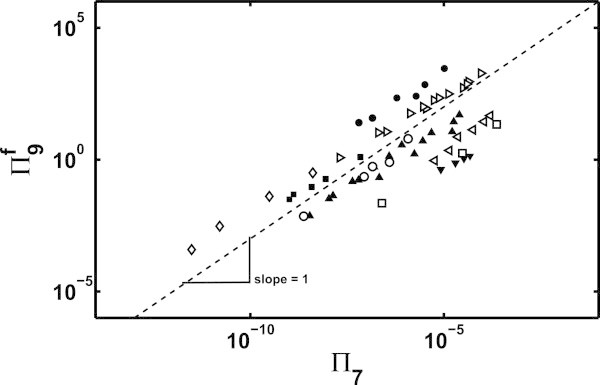
**vs** ***Π***_**7**_ **,** **plotted using experimental data from several papers; the individual symbols represent the following,** ***⊲*** **PA6/FA (Mit-uppatham et al.** 2004 **), ▴ PA-6/FA/DCM (Wei et al.** 2010 **), • PS/THF/LiCI** ***O***_**4**_ **(Wang et al.** 2006 **), ◊ PS-U2 (Wang et al.** 2011 **), ▾ PEO/DNA/Water (Liu et al.** 2007 **), ■ PAN/DMF (Wang et al.** 2007 **),** ***⊳*** **PHB/CF/DMF (Wang et al.** 2008 **), ◦ PAA-Water (Li and Hsiehg** 2005 **) and □ PAA-DMF (Li and Hsiehg** 2005).

It was found that all the data sets in the plot of  vs *Π*_7_ had slope ∼ 1, as shown in Figure 1, which lead to the following relation between the two:
20

Since the final fiber diameter was used in calculating , *d*_*f*_ could be extracted as a function of the remaining parameters. Expanding Eq. (20) and after some re-arrangement we got,
21

Equation (21) gives the scaling of various factors with *d*_*f*_. Viscosity, flow rate, conductivity and tip-to-collector distance are directly proportional while applied voltage is inversely proportional to *d*_*f*_. Qualitatively this scaling matches with the trend observed from experiments for the viscosity, flow rate and applied voltage. Equation (21) predicts a scaling coefficient of 0.5 for both viscosity and flow rate. For viscosity, the predicted scaling coefficient is close to the average scaling predicted from the experimental studies. For flow rate however, the average of the three scaling coefficients obtained from experimental studies is 0.325 (Liu et al. 2007; Mataram et al. 2011; Wang et al. 2008), which is lower than 0.5. For △*V*, *L* and *K* there are very few methodical studies (with experimental data) available in literature to compare the coefficient values.

The force balance for bent jet proposed by Hohman et al. (2001) is only valid for Newtonian solutions. Thus polymer properties such as relaxation time and polymer extensional viscosity are not included in this analysis. There are studies which suggest the significant importance of viscoelastic properties in electrospinning (Thompson et al. 2007; Yu et al. 2006). Polymer molecules are known to undergo coil-stretch transition in extensional flows such as in electrospinning. This leads to an escalation of extensional viscosity of the polymer solution. However it is believed that the polymer extensional viscosity would reach its finite extensibility limit during whipping and thus the polymer solution would behave as a high viscosity Newtonian solution. The ratio of steady state extensional viscosity of the polymer solution to zero shear rate viscosity is termed as the steady state Trouton ratio . The value of  for different polymer-solvent systems would be different. Inclusion of these polymer properties could possibly lead to collapse all experimental data on one master curve and help in deriving an universal correlation for final fiber diameter in electrospinning.

It should be noted however, that even with all the shortcomings this crass analysis has the virtue of utter usefulness and ease. Eq. (21) predicts the correct trend for the three most important parameters that affect the electrospinning process, namely viscosity, flow rate and applied voltage. Also the scaling coefficients predicted are fairly close to the experimental studies. As pointed out, more systematic studies need to be carried out to verify the correctness of the scalings predicted and additional physics needs to be added to arrive at a universal correlation between final fiber diameter and the process parameters of electrospinning.

## References

[CR1] Beachley V, Wen X (2009). Effect of electrospinning parameters on the nanofiber diameter and length. Mater Sci Eng C Mater Biol Appl.

[CR2] Bhattacharjee PK, Schneider TM, Brenner MP, McKinley GH, Rutledge GC (2010). On the measured current in electrospinning. J Appl Phys.

[CR3] Cui W, Li X, Zhou S, Weng J (2007). Investigation on process parameters of electrospinning system through orthogonal experimental design. J Appl Polym Sci.

[CR4] Deitzel JM, Kleinmeyer J, Harris D, Beck Tan NC (2001). The effect of processing variables on the morphology of electrospun nanofibers and textiles. Polymer.

[CR5] Feng JJ (2002). The stretching of an electrified non-newtonian jet: a model for electrospinning. Phys Fluids.

[CR6] Fridrikh S, Yu J, Brenner M, Rutledge G (2003). Controlling the fiber diameter during electrospinning. Phys Rev Lett.

[CR7] Greiner A, Wendorff JH (2008). Functional self-assembled nanofibers by Electrospinning. Adv Polym Sci.

[CR8] Gupta P, Elkins C, Long TE, Wilkes GL (2005). Electrospinning of linear homopolymers of poly(methyl methacrylate): exploring relationships between fiber formation, viscosity, molecular weight and concentration in a good solvent. Polymer.

[CR9] Han D, Steckl AJ (2009). Superhydrophobic and oleophobic fibers by coaxial electrospinning. Langmuir.

[CR10] Helgeson ME, Wagner NJ (2007). A correlation for the diameter of electrospun polymer nanofibers. AIChE J.

[CR11] Helgeson M, Grammatikos K, Deitzel J, Wagner N (2008). Theory and kinematic measurements of the mechanics of stable electrospun polymer jets. Polymer.

[CR12] Hohman MM, Shin M, Rutledge G, Brenner MP (2001). Electrospinning and electrically forced jets. I. stability theory. Phys Fluids.

[CR13] Homayoni H, Ravandi SAH, Valizadeh M (2009). Electrospinning of chitosan nanofibers: Processing optimization. Carbohydr Polymer.

[CR14] Jeun J-P, Kim Y-H, Lim Y-M, Choi J-H, Jung C-H, Kang P-H, Nho Y-C (2007). Electrospinning of poly(l-lactide-co-d, l-lactide). J Ind Eng Chem.

[CR15] Li L, Hsiehg Y-L (2005). Ultra-fine polyelectrolyte fibers from electrospinning of poly(acrylic acid). Polymer.

[CR16] Liu Y, Chen J, Misoska V, Wallace G (2007). Preparation of novel ultrafine fibers based on DNA and poly(ethylene oxide) by electrospinning from aqueous solutions. React Funct Polym.

[CR17] Kim B, Park H, Lee S-H, Sigmund WM (2005). Poly(acrylic acid) nanofibers by electrospinning. Mater Lett.

[CR18] Kirichenko VN, Petryanov-Sokolov IV (1986). Asymptotic radius of a slightly conducting liquid jet in an electric field. Soviet Phys Dokl.

[CR19] Mataram A, Ismail AF, Abdullah MS, Ng BC, Matsuura T (2011). A review of assembled polyacrylonitrile-based carbon nanofiber prepared electrospinning process. Int J Nanosci.

[CR20] McCann JT, Marquez M, Xia Y (2006). Melt coaxial electrospinning: a versatile method for the encapsulation of solid materials and fabrication of phase change nanofibers. Nano Lett.

[CR21] McKee MG, Wilkes GL, Colby RH, Long TE (2004). Correlations of solution rheology with electrospun fiber formation of linear and branched polyesters. Macromol.

[CR22] Mazoochi T, Jabbari V (2011). Chitosan nanofibrous scaffold fabricated via electrospinning: the effect of processing parameters on the nanofiber morphology. Int J Polym Anal Charact.

[CR23] Mit-uppatham C, Nithitanakul M, Supaphol P (2004). Ultrafine electrospun polyamide-6 fibers: effect of solution conditions on morphology and average fiber diameter. Macromol Chem Phys.

[CR24] Mohan A (2002). Formation and characterization of electrospun nonwoven webs.

[CR25] Ramakrishna S, Fujihara K, Teo W, Lim T, Ma Z (2005). An Introduction to Electrospinning and Nanofibres.

[CR26] Reneker DH, Yarin AL, Fong H (2000). Bending instability of electrically charged liquid jets of polymer solutions in electrospinning. J Appl Phys.

[CR27] Rojas OJ, Montero GA, Habibi Y (2009). Electrospun nanocomposites from polystyrene loaded with cellulose nanowhiskers. J Appl Polym Sci.

[CR28] Sajeev US, Anand KA, Menon D, Nair S (2008). Control of nanostructures in pva, pva/chitosan blends and pcl through electrospinning. Bull Mater Sci.

[CR29] Singh S, Lakshmi SG, Vijayakumar M (2009). Effect of process parameters on the microstructural characteristics of electrospun poly(vinyl alcohol) fiber mats. Nanobiotechnology.

[CR30] Supaphol P, Chuangchote S (2008). On the electrospinning of poly(vinyl alcohol) nanofiber mats: a revisit. J Appl Polym Sci.

[CR31] Thompson CJ, Chase GG, Yarin AL, Reneker DH (2007). Effects of parameters on nanofiber diameter determined from electrospinning model. Polymer.

[CR32] Wang C, Cheng Y-W, Hsu C-H, Chien H-S, Tsou S-Y (2011). How to manipulate the electrospinning jet with controlled properties to obtain uniform fibers with the smallest diameter?—a brief discussion of solution electrospinning process. J Polym Res.

[CR33] Wang C, Chien H-S, Hsu C-H, Wang Y-C, Wang C-T, Lu H-A (2007). Electrospinning of polyacrylonitrile solutions at elevated temperatures. Macromol.

[CR34] Wang C, Hsu C-H, Hwang IH (2008). Scaling laws and internal structure for characterizing electrospun poly[(R)-3-hydroxybutyrate] fibers. Polymer.

[CR35] Wang C, Hsu C-H, Lin J-H (2006). Scaling laws in electrospinning of polystyrene solutions. Macromol.

[CR36] Wang C, Yuan J, Niu H, Yan E, Zhao H (2009). Investigation of fundamental parameters affecting electrospun pva/cus composite nanofibres. Pigm Resin Technol.

[CR37] Wannatong L, Sirivat A, Supaphol P (2004). Effects of solvents on electrospun polymeric fibers: preliminary study on polystyrene. Polym Int.

[CR38] Wei W, Yeh J-T, Li P, Li M-R, Li W, Wang X-L (2010). Effect of nonsolvent on morphologies of polyamide 6 electrospun fibers. J Appl Polym Sci.

[CR39] Yarin AL (2011). Coaxial electrospinning and emulsion electrospinning of core-shell fibers. Polymer Adv Tech.

[CR40] Yu D-G, Branford-White C, Bligh SWA, White K, Chatterton NP, Zhu L-M (2011). Improving polymer nanofiber quality using a modified co-axial electrospinning process. Macromol Rapid Comm.

[CR41] Yu JH, Fridrikh SV, Rutledge GC (2006). The role of elasticity in the formation of electrospun fibers. Polymer.

[CR42] Yu D-G, Williams GR, Wang X, Liu XK, Li HL, Bligh SWA (2013). Coaxial electrospinning using a concentric Teflon spinneret to prepare biphasic-release nanofibers of helicid. RSC Adv.

[CR43] Yuan XY, Zhang YY, Dong C, Sheng J (2004). Morphology of ultrafine polysulfone fibers prepared by electrospinning. Polym Int.

[CR44] Zhang Y, Huang Z-M, Xu X, Lim CT, Ramakrishna S (2004). Preparation of core-shell structured pcl-r-gelatin bi-component nanofibers by coaxial electrospinning. Chem Mater.

[CR45] Zhang C, Yuan X, Wu L, Han Y, Sheng J (2005). Study on morphology of electrospun poly(vinyl alcohol) mats. Eur Polym J.

